# Measuring Abnormality in High Dimensional Spaces with Applications in Biomechanical Gait Analysis

**DOI:** 10.1038/s41598-018-33694-3

**Published:** 2018-10-19

**Authors:** Michael Marks, Trevor Kingsbury, Richard Bryant, John David Collins, Marilynn Wyatt

**Affiliations:** 1 Improvement Path Systems, Rochester, MI USA; 2 Naval Medical Center San Diego, San Diego, CA USA

**Keywords:** Rehabilitation, Biomedical engineering, Computational science

## Abstract

Accurately measuring a subject’s abnormality using high dimensional data can empower better outcomes research. Utilizing applications in instrumented gait analysis, this article demonstrates how using data that is inherently non-independent to measure overall abnormality may bias results. A methodology is then introduced to address this bias and accurately measure abnormality in high dimensional spaces. While this methodology is in line with previous literature, it differs in two major ways. Advantageously, it can be applied to datasets in which the number of observations is less than the number of features/variables, and it can be abstracted to practically any number of domains or dimensions. Initial results of these methods show that they can detect known, real-world differences in abnormality between subject groups where established measures could not. This methodology is made freely available via the *abnormality* R package on CRAN.

## Introduction

**Figure 1:**
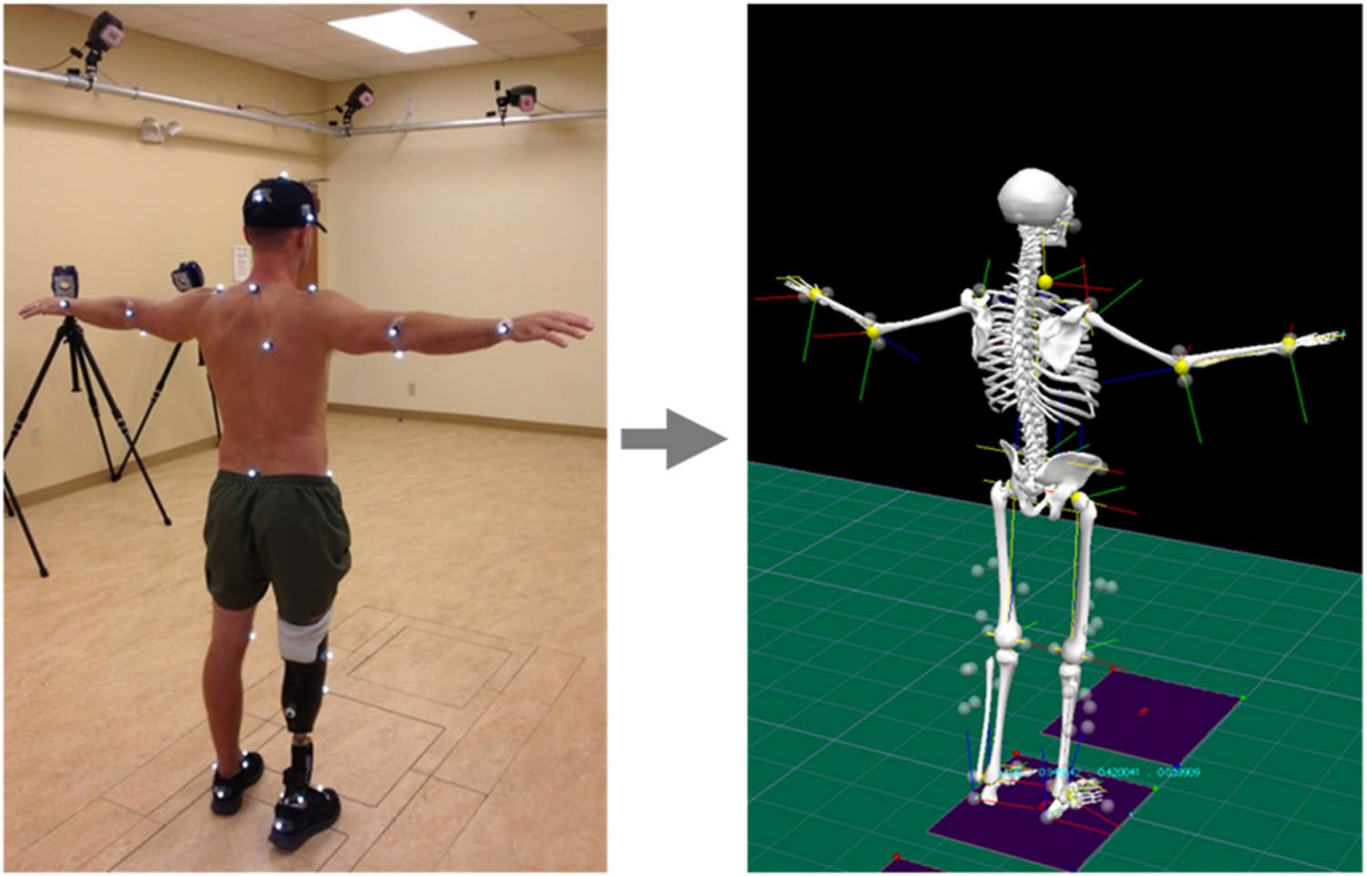
An example of a patient preparing for instrumented gait analysis.

Recent advances in data collection have enabled researchers to collect large amounts of data to describe numerous dimensions (i.e. variables, features, etc.) of their research subjects. Aggregating these many dimensions into a single measure that describes the subject in a meaningful way is often necessary. In the case of instrumented gait analysis (Fig. 1), describing a subject’s overall level of abnormality is meaningful to both researchers and clinicians. Therefore, measuring overall abnormality across the many dimensions of human gait is necessary to empower both clinical decision making and outcomes research.

Instrumented gait analysis has been widely used for a variety of pediatric and adult pathologies as a means of either quantifying a functional movement deficit or evaluating improvements due to rehabilitation treatments^1^. A typical gait data collection yields thousands of unique measurement dimensions that quantify joint position and force production. It is with these thousands of measurement dimensions that gait researchers need to define a patient’s overall abnormality.

Measuring a patient’s overall level of abnormality is typically done by comparing a patient’s gait data to a reference population data-set of able-bodied controls. When this comparison is done in a single measurement dimension, simple distance measures (e.g. Euclidean, Manhattan, etc.) provide unbiased results. However, typically collected gait data consists of thousands of non-independent measurement dimensions. If this dependency structure is not properly addressed, using standard distance measures to define overall abnormality can produce biased results.

The Mahalanobis distance measure^2^ attempts to address this bias, but it cannot be calculated when the number of observations (*n*) is less the number of measurement dimensions (*p*)^3,4^. This is problematic since *p* > *n* for many high-dimensional data-sets, especially those found in instrumented gait analysis. Schutte *et al*.^1^ also attempted to address this bias for a gait normalcy index; however, this measure was made up of only 16 univariate parameters that provide an incomplete picture of overall gait^5^.

Attempting to address this incompleteness, two new gait normalcy indexes were created by Schwartz and Rozumalski^5^ and Baker *et al*.^6^ to measure normalcy across 459 dimensions during a gait cycle. However, these two methodologies use standard distance measures and fail to address the dependency structure of the underlying data. Despite this, the results from these methodologies have become standard gait summary measures reported in various populations^7–13^.

Having an overall gait abnormality measure that accounts for all the dimensions of human gait, while still accounting for the natural dependency between those dimensions, would ensure proper conclusions are drawn in clinical decision making and outcomes research. Therefore, the purpose of this study is to: (1) Demonstrate the inherent non-independent nature of data produced in instrumented gait analysis, (2) illustrate how this dependency structure can bias measures of overall abnormality, and (3) put forth a methodology to accurately measure overall abnormality in high dimensional spaces.

## Results

### The Inherent Non-Independent Nature of Gait Data

**Figure 2:**
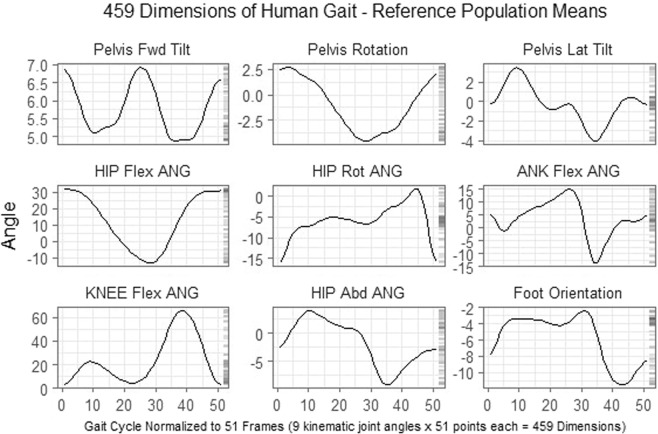
Reference population (n = 32) means for nine joint angles across the gait cycle normalized to 51 points. These 459 total dimensions are the same as those used by Schwartz and Rozumalski^5^ and Baker *et al*.^6^ for their overall gait abnormality measures. This data was aggregated into a 32 × 459 matrix for the multicollinearity analysis.

Human gait is a complex movement that consists of both open and closed kinematic chain movements. While motion is typically analyzed at the joint level, movement of one joint can result in changes at other joints. Thus, gait elements are non-independent and the data representing it will not be either. To demonstrate this non-independence, gait data were collected for 32 able-bodied males and assembled into a 32 × 459 matrix (See Methods section for collection methodology). These are the same 459 dimensions (9 kinematic joint angles × 51 points each) used by Schwartz and Rozumalski^5^ and Baker *et al*.^6^ for their overall gait abnormality measures (Fig. 2).

**Figure 3:**
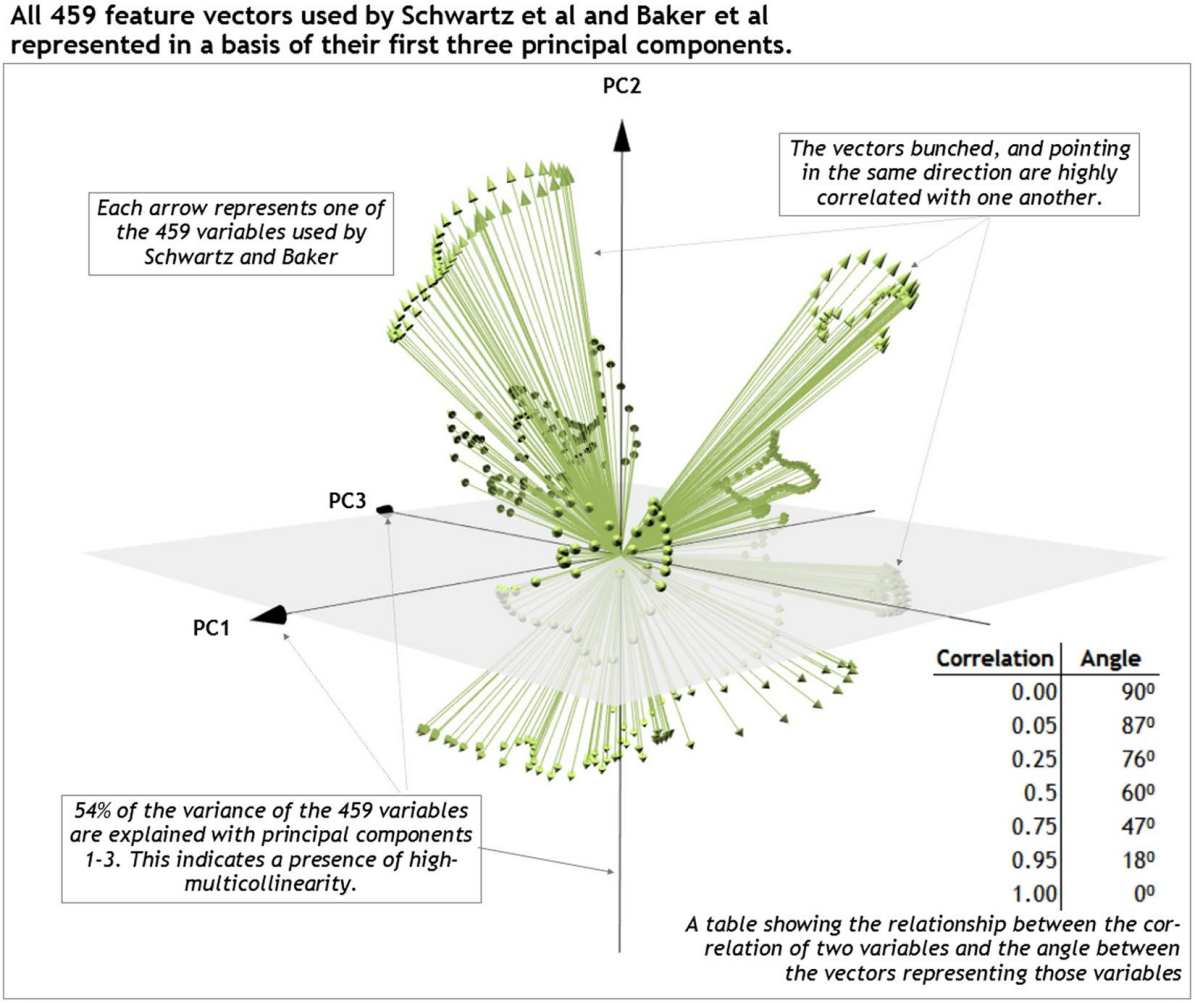
All 459 feature vectors of the reference gait data matrix projected onto their first three eigenvectors (principal components). The non-random distribution of the vectors in this space indicate a strong dependency among the gait features.

By projecting all 459 feature vectors of this reference matrix onto their first three eigenvectors (principal components), the non-independent nature of the gait data can be seen visually (Fig. 3)^14^. There is a clear, non-random pattern to the vectors, demonstrating the dependent nature of the underlying variables they represent.

### The Non-Independence Bias Problem

This dependency structure can bias measures of overall abnormality if not properly addressed. This bias is most easily demonstrated by visually exploring overall abnormality measurements of a subject against a reference population calculated with non-independent variables in two-dimensional space.

**Figure 4:**
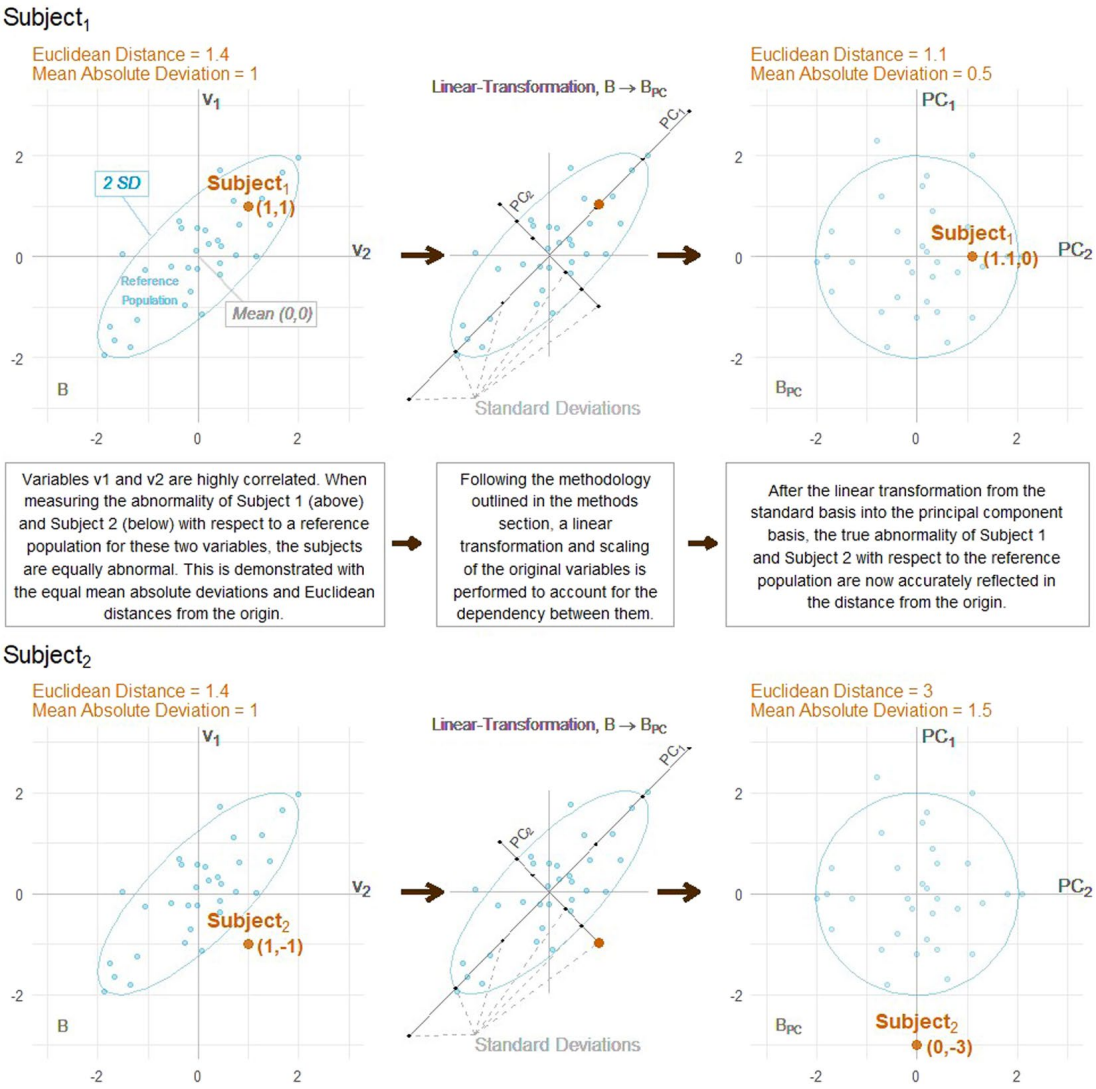
A two-dimensional example of the non-independence bias problem when measuring overall abnormality with two correlated variables (r = 0.83) and how that bias is addressed with the proposed methodology.

The four graphs in Fig. 4 show the values of two correlated variables (*v*_1_ and *v*_2_, *r* = 0.83) for a reference population (blue dots, *n* = 32) and two different subjects (orange), *Subject*_1_ and *Subject*_2_. On the left, the variables are in their scaled and centered form (*μ* = 0, *ρ* = 1). We will refer to this basis as the standard basis $B=\{{\hat{b}}_{1},\,{\hat{b}}_{2},\,\ldots ,\,{\hat{b}}_{p}\}$. On the right, *v*_1_ and *v*_2_ are transformed into a basis of their orthonormal principal component vectors utilizing the methodology laid out in the methods section. We will refer to this basis as the principal component basis ${B}_{PC}=\{{\widehat{PC}}_{1},\,{\widehat{PC}}_{2},\,\ldots ,\,{\widehat{PC}}_{p}\}$. Combinations of *v*_1_ and *v*_2_, and *PC*_1_ and *PC*_2_, that are within two standard deviations of the mean of the reference population will be inside the blue ellipse. Since the data have been centered and scaled (*μ* = 0, *ρ* = 1), the mean reference subject lies at the origin for both graphs.

The Euclidean distance between the subject and the origin represents the level of abnormality. When comparing the Euclidean distances of *Subject*_1_ and *Subject*_2_, the issues with measuring normalcy in the standard basis, *B*, can be seen. In *B* (Fig. 4), both *Subject*_1_ and *Subject*_2_ are $\sqrt{2}$ units from the origin, indicating equal abnormality. However, due to the strong positive correlation between *v*_1_ and *v*_2_, *Subject*_2_’s *combination* of the two variables is more abnormal than *Subject*_1_’s. This is demonstrated by comparing locations in relation to the blue ellipse representing two standard deviations from the origin. This interesting example shows that not addressing multicollinearity can lead to biased results and how the proposed methodology negates this bias.

**Figure 5:**
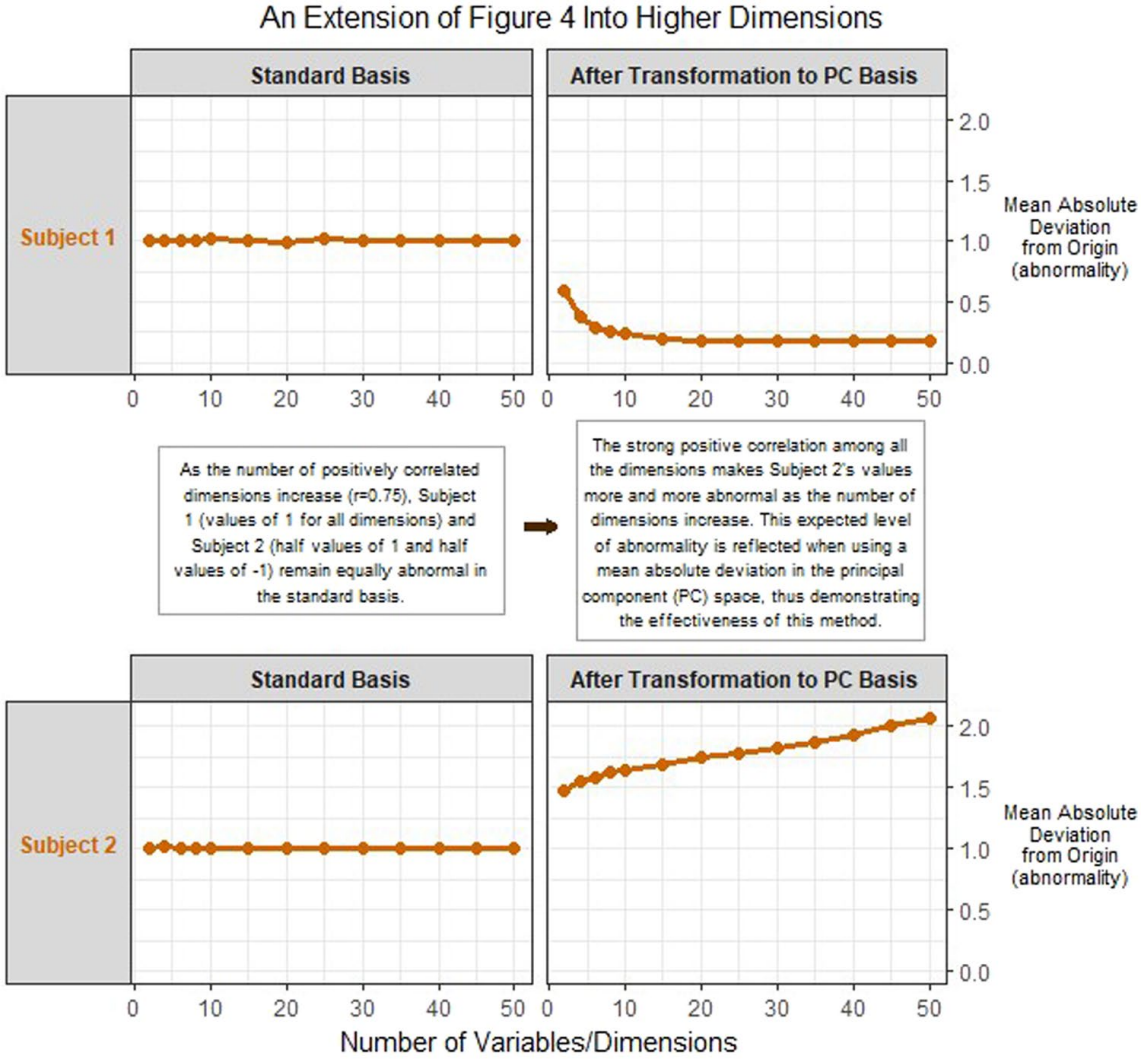
The results of a simulated extension of the example from Fig. 4 into higher dimensional spaces. The results show that the bias when measuring overall abnormality can actually increase as the number of dimensions increase and the true abnormality is reflected when using the proposed methodology.

This example can be extended into higher dimensions as well. Figure 5 shows a simulated extension of the Fig. 4 example into higher dimensional spaces (Methods - Simulated Example of Bias in Higher Dimensional Spaces). This simulated example shows that the bias seen in Fig. 4 actually gets larger as the number of dimensions increase. When using a mean absolute deviation (MAD) in the standard basis, Subject 1 (all values of 1) and Subject 2 (half values of 1 and half values of −1) share the same level of normalcy in any number of dimensions. This is despite a reference population whose features are all positively correlated (r = 0.75). This strong positive correlation makes Subject 2’s values more and more abnormal as the number of dimensions increase. This expected level of abnormality is reflected when using a mean absolute deviation in the principal component (PC) space, thus demonstrating the effectiveness of this method.

As seen by the examples in Figs 4 and 5, not addressing the non-independent nature of the underlying data in a measure of overall abnormality can lead to biased results. This bias only gets worse as the correlation between the underlying variables increases (Supplemental Figure). However, as seen by these results, utilizing the methodology laid out to transform the original variables into a basis of their orthonormal principal component vectors negates this bias.

### Applications in Biomechanical Gait Analysis

**Figure 6:**
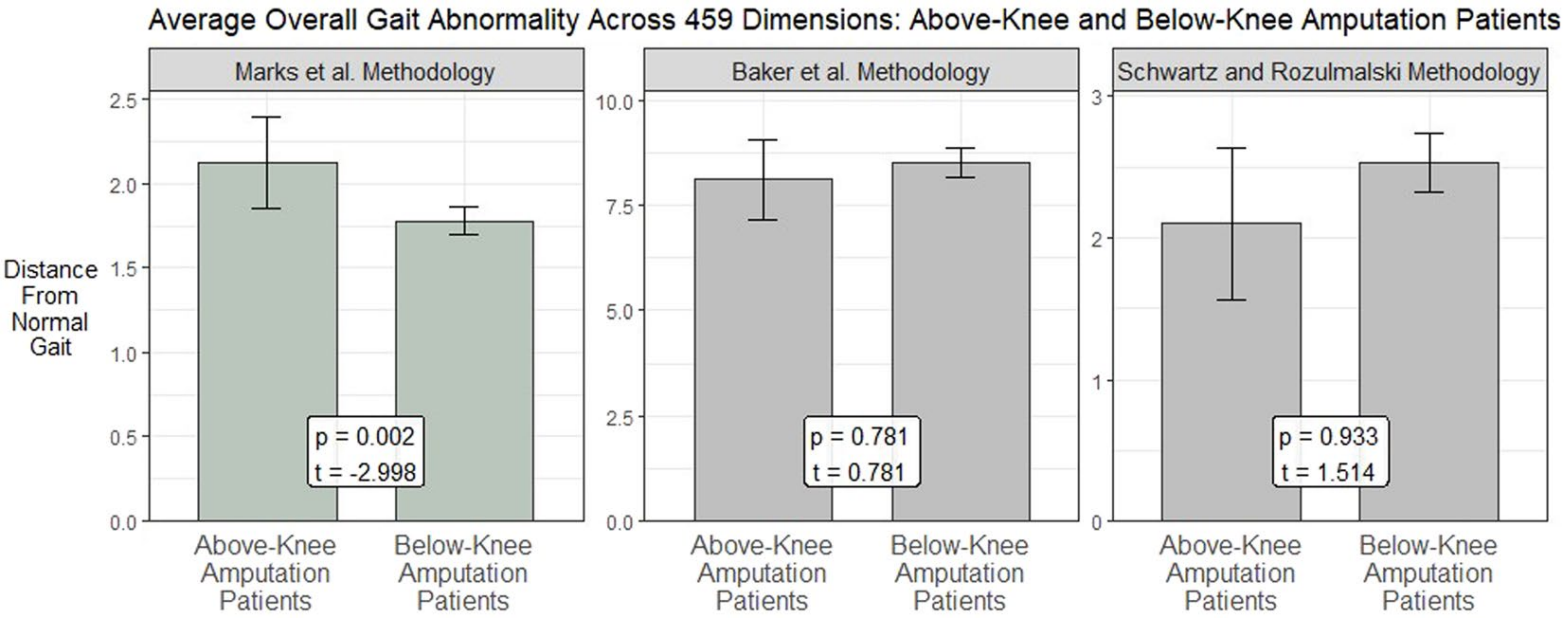
The results of measuring overall gait abnormality utilizing the methodology proposed in this paper and established methodologies in gait research. The average abnormality is shown for two groups with known gait differences: unilateral above-knee amputation patients (n = 10) and unilateral below-knee amputation patients (n = 63). This paper’s proposed methodology is able to detect the known differences between the two groups across these dimensions, while the established methodologies cannot.

Research has shown that patients with an above knee amputation have more abnormal gait than patients with a below knee amputation for kinematic measures such as knee flexion^15^. An overall abnormality measure should be able to detect this difference. Figure 6 shows the average overall abnormality for these two patient groups using different overall abnormality methods. The known differences in gait abnormality between the groups were detected using the methods outlined in this paper (p = 0.002, power = 0.885 at *α* = 0.05). The methodologies that do not account for the non-independent nature of the underlying data were unable to detect these differences. These results demonstrate that accounting for multicollinearity when measuring overall abnormality enables a more accurate measurement.

## Discussion

The purpose of this study was to demonstrate the inherent non-independent nature of data produced in instrumented gait analysis, illustrate how this non-independence can bias measures of overall abnormality, and put forth a methodology to produce a new coordinate system with which to accurately measure overall abnormality in high dimensional spaces. In addition to the new coordinate system, the number of principal components to retain and the proper distance measure to utilize are important discussion topics. The two applications depend on the specific clinical or research question but have several validated options available.

### Determining the Number of Principal Components

Selecting the right number of principal components is an extremely well researched topic (Jackson^16^, Peres-Neto *et al*.^17^, and Ferré^18^ provide good surveys). The extensive research done on this problem speaks to its importance: if the number of axes is not correctly estimated, one can introduce noise (overestimation) or loss of information (underestimation) in the analysis^19^.

In the case of this study’s gait data (and many other high dimensional datasets), there are fewer observations (*n* = 32) than measurement dimensions (*p* = 459). If *n* is smaller than *p*, the data cannot occupy more than an *n*-dimensional subspace of the *p*-dimensional space. Therefore, projection into a lower-dimensional space does not necessarily lose information. If the data really are a lower-dimensional manifold in the high dimensional space, then a projection can preserve its structure exactly^20^. This is the case with our example gait data-set as illustrated by the first 32 principal components accounting for 100% of the total variation in the data (Table 1).

**Table 1:** A breakdown of the first 32 Principal Components for the 459 Dimension Gait Data Sample.

Component Number	Eigenvalue	% Variance Explained	Cumulative %
1	129.96	0.2831	0.2831
2	67.59	0.1473	0.4304
3	52.57	0.1145	0.5449
4	36.59	0.0797	0.6246
5	34.56	0.0753	0.6999
6	30.43	0.0663	0.7662
7	23.10	0.0503	0.8165
8	15.77	0.0343	0.8509
9	12.05	0.0262	0.8771
10	9.35	0.0204	0.8975
11	7.50	0.0163	0.9139
12	6.88	0.0150	0.9288
13	4.87	0.0106	0.9394
14	4.43	0.0097	0.9491
15	4.14	0.0090	0.9581
16	3.39	0.0074	0.9655
17	2.47	0.0054	0.9709
18	2.40	0.0052	0.9761
19	2.05	0.0045	0.9806
20	1.47	0.0032	0.9838
21	1.46	0.0032	0.9870
22	1.32	0.0029	0.9899
23	0.89	0.0019	0.9918
24	0.89	0.0019	0.9937
25	0.72	0.0016	0.9953
26	0.61	0.0013	0.9966
27	0.42	0.0009	0.9976
28	0.37	0.0008	0.9984
29	0.33	0.0007	0.9991
30	0.25	0.0006	0.9996
31	0.16	0.0004	1.0000
32	0.00	0.0000	1.0000

One could choose to retain all these principal components for measuring overall abnormality. This is what was done by Schutte *et al*.^1^ for their gait normalcy index; however, the data used in that study only had 16 dimensions. Keeping all the principal components removes all risk that information relevant to a subject’s abnormality is lost. However, including principal components with small eigenvalues may introduce unnecessary noise (e.g. measurement error) that could bias results. Furthermore, utilizing fewer principal components may be beneficial when making clinical interpretations since one could determine what data are represented in each PC and determine the most meaningful data that is being evaluated.

**Figure 7:**
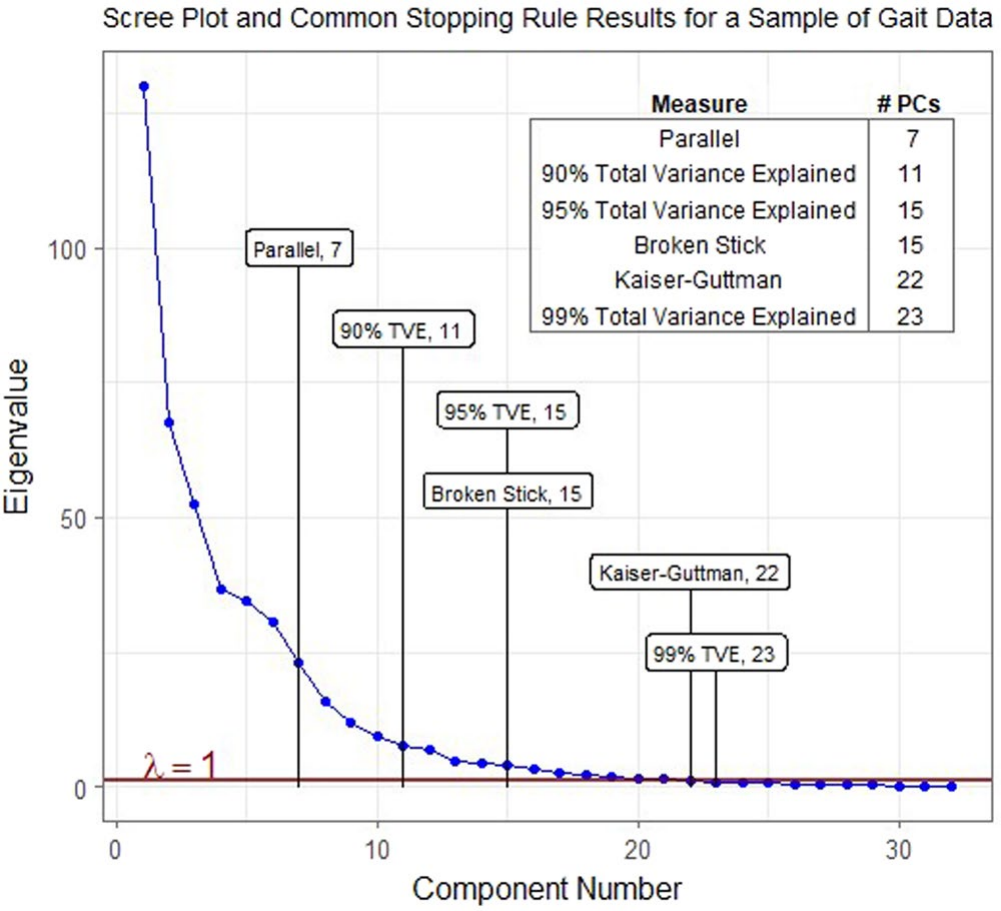
A scree plot with the results of some common methodologies used to determine the appropriate number of principal components when applied to the sample gait data.

Deciding how many of those principal components (in this case 32) to keep is more of a subjective art than a perfect science. Figure 7 shows a scree plot with the results of some common methodologies used to determine the appropriate number of principal components when applied to the sample gait data. Percent total variance explained (% TVE) is a common, yet relatively arbitrary, method; three different cutoff values are presented (90%, 95%, and 99%). Additionally, the results of the broken stick^21^, Kaiser-Guttman^22,23^, and parallel^24^ methods are included as well^25^.

For purely clinical applications, interest lies in utilizing a summary measure to enable specific interventions. To do this, some knowledge of why a subject is abnormal is necessary. Assessing the loading significance of the original variables on the resultant principal components^26^ can enable a clinician to see where a patient is most abnormal. This task becomes more complex with more principal components, so using fewer PCs would enable some clinical interpretability. As a result, even though a lower amount of variance would be explained, the clinical meaning would be improved with fewer principal components.

For the purposes of measuring abnormality in high dimensional gait analysis, it is likely that removing principal components with eigenvalues <1 (Kaiser-Guttman) strikes a good balance between the risks of unnecessary noise and information loss. An eigenvalue greater than one indicates that its corresponding principal component contains more information than any single original variable^16^. If an eigenvalue equals one for a given principal component, that variability could be associated exclusively with one single original variable that is orthogonal to the rest of the data. While this scenario is highly unlikely, one would probably not want to exclude a variable which exhibits these properties for the purposes of abnormality measurement. Keeping all principal components with an eigenvalue >1 would ensure this does not happen.

An additional consideration is that for very high dimensional datasets with *p* much larger than *n*, the eigenvalues from the data are not consistent estimators of the population eigenvalues^27^. Therefore, caution should be exercised before drawing conclusions from the eigenvalues or TVEs from the sample. A possible approach in this circumstance is to use bootstrapping. This allows one to create a confidence interval either of the true eigenvalue corresponding to each principal component, or the percentage of variance explained by the first k PCs, through resampling the data. This confidence interval can be used to test, at a specified confidence level, that an eigenvalue <1 or TVE is 90%. Therefore, the first PC that satisfies this test can be used as the cutoff point for PCs to retain. To maintain a balance between clinical interpretability and variance explained as well as keep the procedure computationally simple, we recommend the Kaiser-Guttman approach to selecting number of principal components, with bootstrapping in cases of *p* being much greater than *n*.

### Selecting a Distance Measure

Measuring distances and the properties of different distance measures in high-dimensional spaces is a well-studied topic for the purposes of outlier detection in computer science^28–33^. However, computer scientists are typically more concerned with how points relate to one another in terms of distance, instead of normalcy which would simply be distance from average (i.e. the origin if the variables are standardized). Further research is required to truly understand the implications of measuring distance from the origin in these higher dimensional spaces.

Established measures of overall gait abnormality use various distance measures in their methodologies. Schutte *et al*.^1^ uses the square of a Euclidean distance. Schwartz and Rozumalski^5^ use the natural logarithm of a Euclidean distance as a raw score, and utilize a scaled version (*μ* = 100, *σ* = 10) of the raw score for clinical interpretation purposes. Baker *et al*.^6^ utilize a simple root mean squared (RMS) difference.

Any number of distance measures (e.g. Euclidean, MAD, RMS, Manhattan, etc.) all have their merit, but ultimately, the metric used to measure distance should be chosen based on the use case. If the resultant abnormality measure is for statistical tests, one would want a measure whose output has good mathematical properties (e.g. normally distributed so parametric tests can be used). This was the rationale for Schwartz and Rozumalski^5^ using the natural logarithm of a Euclidean distance. If it is for interpretation purposes, one would want something the user of the data will understand (the rationale for Schwartz and Rozumalski’s scaled version^5^). Ideally, usability testing would be done to establish a unit of measurement that is most valuable to its users. For example, if the users are familiar with the idea of a standard deviation, a mean absolute deviation (MAD) of the standardized principal components could be used. This would improve clinical interpretability and likely enhance utility in a multidisciplinary setting.

## Conclusion

Biomechanical gait analysis is a powerful tool for collecting large amounts of outcomes data. Aggregating all this data into a single measure of abnormality greatly aids in clinical decision making and outcomes research. As a result, previous attempts at creating such measures have become widely used in many academic and clinical circles^5,6^. These measures have been a big step forward for the gait community; however, as this study has demonstrated, these measures can bias results because they fail to address the inherent dependent nature of gait data.

This study has given the researcher a methodology to address these dependency issues when creating overall abnormality measures. These methods are not exclusive to measuring abnormality in gait and could be applied to other high-dimensional, multicollinear data-sets. Given each researcher/clinician has their own needs when using or creating outcome measures, different considerations can be utilized that will affect the resultant measure. Application of these methods and considerations will empower researchers to create useful measures of overall abnormality in domains like instrumented gait analysis. Utilizing these new measures will improve the accuracy of outcomes research in such fields where multicollinear, high-dimensional datasets exist.

## Methods

### Subject Data Collection

Thirty-two able-bodied males, with no history of orthopedic injuries or surgeries that would affect gait, were studied while walking at their self-selected speed. The average age of these subjects was 30 ± 6 years, and their average BMI was 27 ± 2 kg/m^2^. The study protocol was approved by the Naval Medical Center San Diego Institutional Review Board in compliance with all applicable federal regulations governing the protection of human subjects. Informed consent was obtained from all subjects who participated in the study. Additionally, informed consent was obtained from the subject in Fig. 1 to have their photo disseminated in the public domain, this includes an open-access publication.

All subjects were studied using a 34-marker modified Helen Hayes marker set and data were collected using a 12-camera Motion Analysis Eagle system (Motion Analysis Corporation, Santa Rosa, CA). This marker set allowed for nine joint angles (pelvic and hip angles in all three planes, knee flex/extension, ankle dorsi/plantarflexion, and foot progression) to be calculated at 2% increments throughout the entire gait cycle of 100%, giving 51 data points per joint angle. The analysis of the volunteer’s left side only resulted in 459 total measurement dimensions for each subject with which to measure normalcy (9 angles × 51 points each = 459 dimensions) (Fig. 2). To mirror the data used by Schwartz and Rozumalski^5^ and Baker *et al*.^6^, the exported data on these subjects were aggregated into a 32 × 459 matrix for the multicollinearity analysis.

### Methodology to Address the Non-Independence Bias Problem

To address the multicollinearity bias problem to accurately measure normalcy in *p* dimensions:

Let *Ref*^*n* × *p*^ be the matrix representing the reference population.

$$Re{f}^{n\times p}=(\begin{array}{ccccc}{r}_{11} & | & | &  & {r}_{1p}\\ \vdots  & {\overrightarrow{r}}_{2} & {\overrightarrow{r}}_{3} & \cdots  & \vdots \\ {r}_{n1} & | & | &  & {r}_{np}\end{array})\in {{\mathbb{R}}}^{nxp}$$

The rows of the matrix represent *n* subjects in the reference population; the columns represent *p* features of the subjects in the reference population.

Let $\overrightarrow{{\bf{S}}{\bf{u}}{\bf{b}}{\bf{j}}}$ be the vector of those same *p* features for the subject with whom we want to compare to the reference population.

$$\overrightarrow{{\bf{S}}{\bf{u}}{\bf{b}}{\bf{j}}}=({s}_{1},\,{s}_{2},\,\ldots ,\,{s}_{p})\text{'}\in {{\mathbb{R}}}^{p}$$

To get these two objects into the principal component basis, *B*_*PC*_, we must first scale and center both based on ${\overrightarrow{\mu }}_{ref}$ and ${\overrightarrow{\sigma }}_{ref}$ where:

$${\overrightarrow{\mu }}_{ref}=({\mu }_{1},\,{\mu }_{2},\,\ldots ,\,{\mu }_{p})^{\prime} \in {{\mathbb{R}}}^{p}$$

and the *p*th element of ${\overrightarrow{\mu }}_{ref}$ is:

$${\mu }_{p}=\frac{1}{n}\sum _{i=1}^{n}Re{f}_{i,p}$$

And where:

$${\overrightarrow{\sigma }}_{ref}=({\sigma }_{1},\,{\sigma }_{2},\,\ldots ,\,{\sigma }_{p})$$

where the *p*th element of ${\overrightarrow{\sigma }}_{ref}$ is:

$${\sigma }_{p}=\sqrt{\frac{{\sum }_{i=1}^{n}{(Re{f}_{i,p}-{\mu }_{p})}^{2}}{n-1}}$$

To get the scaled and centered version of $Re{f}^{n\times p}$ (We will call this $Re{f}_{Z}^{n\times p}$), ${\overrightarrow{\mu }}_{ref}$ is subtracted from all *n* rows of $Re{f}^{n\times p}$, all *n* rows are then divided by ${\overrightarrow{\sigma }}_{ref}$. The same process is done on $\overrightarrow{{\bf{S}}{\bf{u}}{\bf{b}}{\bf{j}}}$ to convert it to ${\overrightarrow{{\bf{S}}{\bf{u}}{\bf{b}}{\bf{j}}}}_{Z}$:

$${\overrightarrow{{\bf{S}}{\bf{u}}{\bf{b}}{\bf{j}}}}_{Z}=\frac{\overrightarrow{{\bf{S}}{\bf{u}}{\bf{b}}{\bf{j}}}-{\overrightarrow{\mu }}_{ref}}{{\overrightarrow{\sigma }}_{ref}}$$

To determine the change of basis matrix, let ${{\bf{E}}}_{B\to {B}_{PC}}$ represent the change of basis matrix from basis *B* to basis *B*_*PC*_. ${{\bf{E}}}_{B\to {B}_{PC}}$ which is simply composed of the eigenvectors of the covariance matrix of ${\bf{R}}{\bf{e}}{{\bf{f}}}_{n,p}^{Z}$. Since the covariance matrix is symmetric, its eigenvectors are orthogonal, thus using a matrix of these eigenvectors as a change of basis matrix results in a rotation of the original data. A change of location (or translation) by scaling and centering followed by a rotation does not alter the intrinsic statistical properties of the data^34^. The overall goal of this transformation is to create a new set of uncorrelated variables with which to measure the distinct properties of the reference population and how a subject differs from that population.

We will refer to the results of the projection of $Re{f}_{Z}^{n\times p}$ and ${\overrightarrow{{\bf{S}}{\bf{u}}{\bf{b}}{\bf{j}}}}_{Z}$ onto the eigenvectors of $cov(Re{f}_{Z}^{n\times p})$ as, $Re{f}_{PC}^{n\times p}$ and ${\overrightarrow{{\bf{S}}{\bf{u}}{\bf{b}}{\bf{j}}}}_{PC}$ respectively.

**Figure 8:**
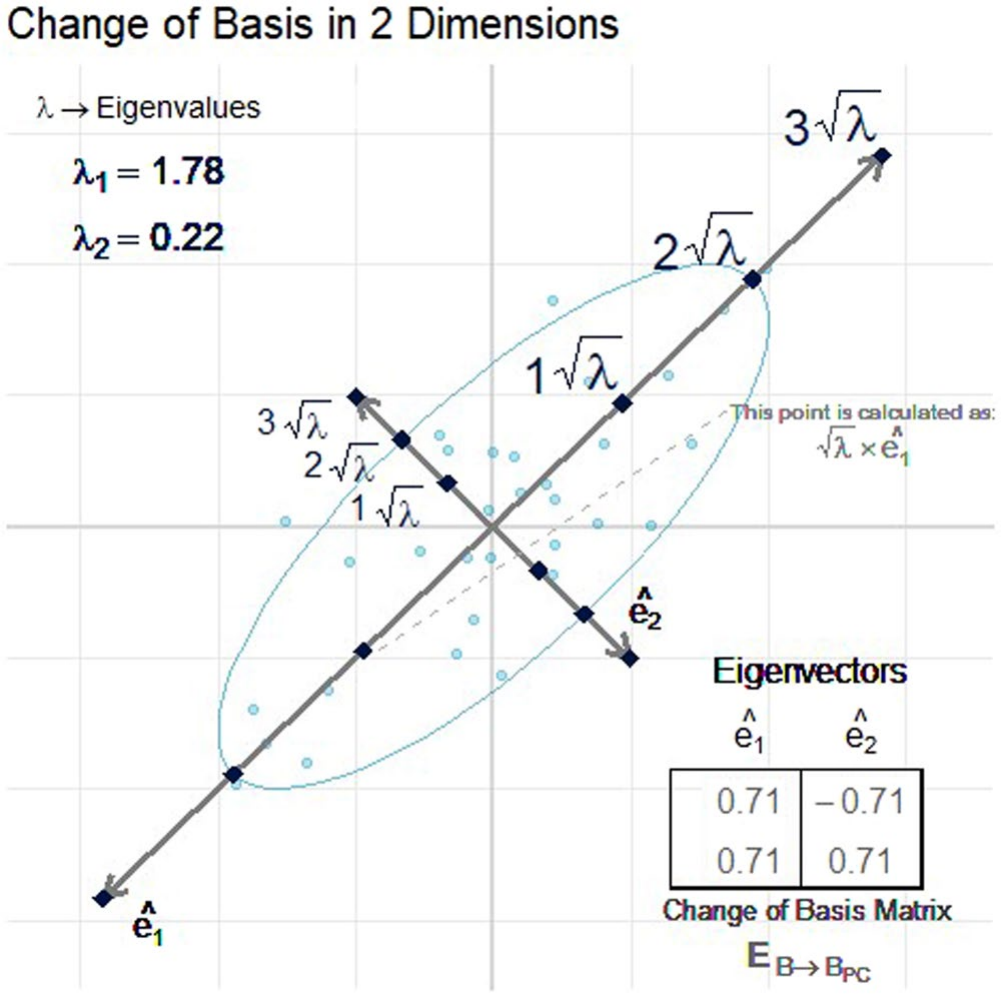
A two-dimensional example of the scaling that is done to ensure that each of the new uncorrelated variables has equal variance. This is done by dividing each new variable by the square root of its corresponding eigenvalue. The square root of the eigenvalue is the standard deviation of its corresponding eigenvector.

The square root of the eigenvalue is the standard deviation of its corresponding eigenvector, so each new uncorrelated variable is divided by the square root of its corresponding eigenvalue to ensure equal variance (Fig. 8). According to Schutte *et al*.^1^, scaling the new variables this way accounts for the magnitude of variation inherent in certain variables. In other words, if one of the original *p* variables (or some linear combination of the *p* variables) has a large variation within the reference population, then a large deviation from the average value of that variable will not count excessively against the ‘normalcy’ of a subject. It could be argued that the eigenvectors associated with small eigenvalues represent variable combinations that may be small random fluctuations and should not be magnified through division by their eigenvalue^1^. This is a valid concern which could be addressed by removing principal components with small eigenvalues from the analysis altogether. This is a common practice and its merits are considered in the discussion section.

The re-scaled versions of $Re{f}_{PC}^{n\times p}$ and ${\overrightarrow{{\bf{S}}{\bf{u}}{\bf{b}}{\bf{j}}}}_{PC}$ will be referred to as $Re{f}_{P{C}_{Z}}^{n\times p}$ and ${\overrightarrow{{\bf{S}}{\bf{u}}{\bf{b}}{\bf{j}}}}_{P{C}_{Z}}$. It is with respect to these variables that normalcy can be accurately measured.

These new variables are a different representation of the original points defining a subject in space. The original points have not changed their location in space, but only the construct in which they are expressed has changed.

Since $Re{f}_{P{C}_{Z}}^{n\times p}$ and ${\overrightarrow{{\bf{S}}{\bf{u}}{\bf{b}}{\bf{j}}}}_{P{C}_{Z}}$ have been scaled and centered, the mean for each variable lies at the origin (i.e. $(0,0,\mathrm{...},{0}_{p})$) of the *p* dimensional space in which they occupy. The distance from the origin can therefore be thought of as a level of abnormality. The further away a subject is from the origin, the more abnormal they are.

### Simulated Example of Bias in Higher Dimensional Spaces

Let ${\Sigma }^{p\times p}$ be the matrix representing the covariance matrix used to generate a reference population ($Re{f}^{100\times p}$) from a multivariate normal distribution^35^ with *μ* = 0.

$${\Sigma }^{p\times p}=(\begin{array}{cccc}1 & {0.75}_{1,2} & \cdots  & {0.75}_{1,p}\\ {0.75}_{2,1} & 1 & \ddots  & \vdots \\ \vdots  & \ddots  & \ddots  & {0.75}_{p-1,p}\\ {0.75}_{p,1} & \cdots  & {0.75}_{p,p-1} & 1\end{array})$$

For each simulation iteration, a reference population, $Re{f}^{100\times p}$, is generated from ${\Sigma }^{p\times p}$. A subject $\overrightarrow{{\bf{S}}{\bf{u}}{\bf{b}}{\bf{j}}}=({s}_{1},\,{s}_{2},\,\ldots ,\,{s}_{p})^{\prime}$ is then generated as either $\overrightarrow{{\bf{S}}{\bf{u}}{\bf{b}}{{\bf{j}}}_{1}}=(1,1,\ldots ,{1}_{p})$ or $\overrightarrow{{\bf{S}}{\bf{u}}{\bf{b}}{{\bf{j}}}_{2}}=({s}_{1},{s}_{2},\ldots ,{s}_{p})\text{'}\in \{1,-1\}$ with a random distribution of $\frac{p}{2}$ values equal to 1 and $\frac{p}{2}$ values equal to −1.

The Euclidean distance and mean absolute deviation are taken between $Re{f}^{100\times p}$ and $\overrightarrow{{\bf{S}}{\bf{u}}{\bf{b}}{{\bf{j}}}_{1}}$, and $Re{f}^{100\times p}$ and $\overrightarrow{{\bf{S}}{\bf{u}}{\bf{b}}{{\bf{j}}}_{2}}$. These distance measures are taken in both the standard basis, and the principal component basis (following the methodology outlined and utilizing all principal components).

The random sampling of $Re{f}^{100\times p}$, $\overrightarrow{{\bf{S}}{\bf{u}}{\bf{b}}{{\bf{j}}}_{1}}$, and $\overrightarrow{{\bf{S}}{\bf{u}}{\bf{b}}{{\bf{j}}}_{2}}$ and the subsequent abnormality calculations (Euclidean and MAD in standard and PC basis) were done 500 times for each value of *p* (2, 4, 6, 8, 10, 15, 20, 25, 30, 35, 40, 45, 50). Results were averaged for each value of *p* and reported as seen in Fig. 5.

### Applications in Biomechanical Gait Analysis Experiment

A group of 73 patients with amputation (63 below knee, and 10 above knee) were studied as soon as they could ambulate without an assistive device after their amputation. Data was collected using the same methodology outlined above for the able-bodied patients. Measurements were taken on the patient’s affected side using the same 459 gait dimensions used by Schwartz and Rozumalski^5^ and Baker *et al*.^6^. Abnormality measurements were then calculated for each patient using the methods outlined in this paper, those by Schwartz and Rozumalski^5^, and those by Baker *et al*.^6^.

The reported metrics generated with the methods outlined in this manuscript used the Kaiser-Guttman^22^ criteria for selecting the appropriate number of principal components and a mean absolute deviation for a distance measure. Results using Schwartz and Rozumalski’s methodology^5^ are reported in their raw, z-score format; this was done for interpretability and does not affect the conclusions drawn in this experiment.

Effect size was defined by a bias-corrected Hedge’s *g* statistic. At an effect size of 0.977 and a significance level of 0.05, the power of this test was 0.885. All data was tested for violations of the assumptions of a t-test: homogeneity of variance and samples from a normally distributed population. The reference population data was also tested for being sampled from a multivariate Gaussian population using Royston’s test^36,37^, resulting in a failure to reject the null hypothesis that the data was drawn from a multivariate Gaussian population (p = 0.06). Further tests should be done to validate these methods in other multivariate populations.

### Disclaimer

The views expressed in the article are those of the authors and do not reflect the official policy of Department of the Navy, Department of the Army, Department of Defense, or the US Government.

## Electronic supplementary material

1) A visual example of the relationship between correlation, eigenvalues, and overall abnormality, 2) The Mean Kinematic Gait Angles for Trans-Femoral and Trans-Tibial Amputation Subject Population Supplementary Information

## Data Availability

This article was written with RMarkdown^38,39^. All source data and code to reproduce the entire manuscript are organized into an R Project^38,40^ and are freely available at https://github.com/ImprovementPathSystems/Measuring_Abnormality_in_High_Dimensional_Spaces. The methodology is also made available via the *abnormality* R package on CRAN^41^.
