# Supplementary material for: Measuring Abnormality in High Dimensional Spaces with Applications in Biomechanical Gait Analysis
Source: Sci Rep. 2018 Oct 19;8:15481. doi: 10.1038/s41598-018-33694-3 (PMC6195542; doi:10.1038/s41598-018-33694-3)
Supplement: Supplementary file 1 — 1) A visual example of the relationship between correlation, eigenvalues, and overall abnormality, 2) The Mean Kinematic Gait Angles for Trans-Femoral and Trans-Tibial Amputation Subject Population Supplementary Information [file 41598_2018_33694_MOESM1_ESM.docx]

**Measuring Abnormality in High Dimensional Spaces with Applications in Biomechanical Gait Analysis**

Michael Marks*^1^, Trevor Kingsbury^2^, Richard Bryant^1^, John David Collins^2^, Marilynn Wyatt^2^

^1^Improvement Path Systems, Rochester, MI

^2^Naval Medical Center San Diego, San Diego, CA

#
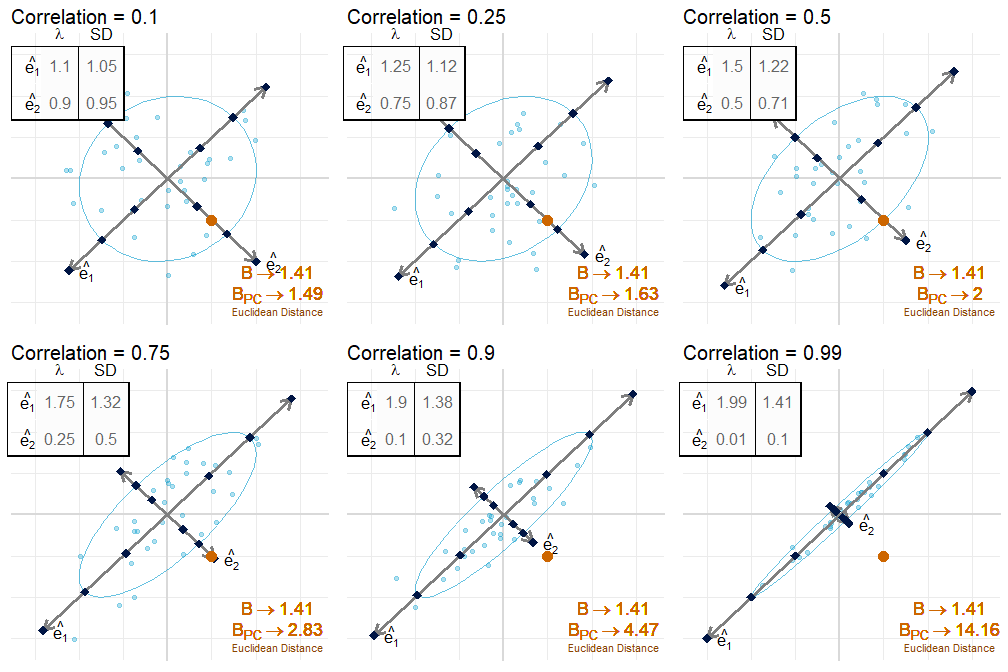
Supplementary Material

Supplemental Figure 1: A visual example of the relationship between correlation, eigenvalues and overall abnormality measurement. As correlation between the two variables increases, the difference in overall abnormality (i.e. Euclidean distance from the origin) between the standard basis and the principal component basis for the subject (orange) grows. This shows that the bias introduced when measuring overall abnormality in the standard basis increases as correlation increases.


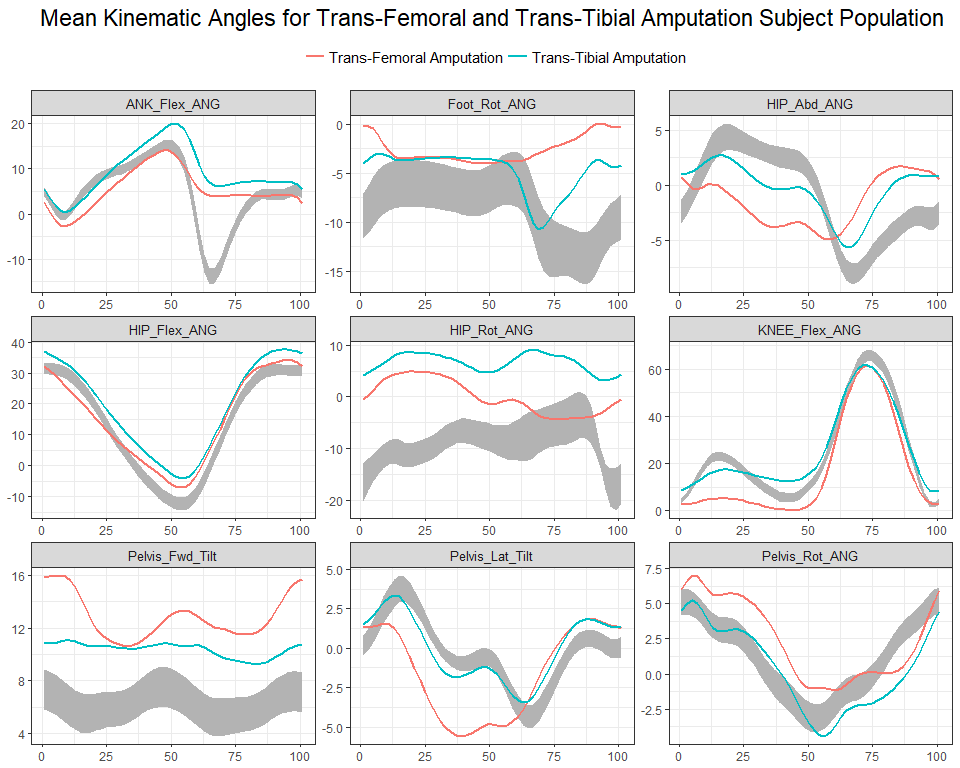
Supplemental Figure 2: The average kinematic gait angles, as compared to the reference population (grey bands, mean +- 1 SD), for two groups: unilateral above-knee amputation patients (n=10) and unilateral below-knee amputation patients (n=63).
